# Characterization of Microcrystalline Cellulose Isolated from Conocarpus Fiber

**DOI:** 10.3390/polym12122926

**Published:** 2020-12-07

**Authors:** H. Fouad, Lau Kia Kian, Mohammad Jawaid, Majed D. Alotaibi, Othman Y. Alothman, Mohamed Hashem

**Affiliations:** 1 Applied Medical Science Department, Community College, King Saud University, P.O. Box 10219, Riyadh 11433, Saudi Arabia; menhfef@ksu.edu.sa; 2 Biomedical Engineering Department, Faculty of Engineering, Helwan University, Cairo 11795, Egypt; 3 Institute of Tropical Forestry and Forest Products (INTROP), Universiti Putra Malaysia (UPM), Serdang 43400, Malaysia; laukiakian@gmail.com; 4 Life Science and Environment Research Institute, King Abdulaziz City for Science and Technology (KACST), Riyadh 11442, Saudi Arabia; mdalotaibi@kacst.edu.sa; 5 Department of Chemical Engineering, College of Engineering, King Saud University, P.O. Box 22452, Riyadh 11451, Saudi Arabia; 6 College of Applied Medical Sciences, King Saud University, P.O. Box 10219, Riyadh 11433, Saudi Arabia; mihashem@ksu.edu.sa

**Keywords:** microcrystalline cellulose, Conocarpus fiber, morphology, crystallinity, thermal stability

## Abstract

Conocarpus fiber is an abundantly available and sustainable cellulosic biomass. With its richness in cellulose content, it is potentially used for manufacturing microcrystalline cellulose (MCC), a cellulose derivative product with versatile industrial applications. In this work, different samples of bleached fiber (CPBLH), alkali-treated fiber (CPAKL), and acid-treated fiber (CPMCC) were produced from Conocarpus through integrated chemical process of bleaching, alkaline cooking, and acid hydrolysis, respectively. Characterizations of samples were carried out with Scanning Electron Microscope (SEM), Energy Dispersive X-ray (EDX), Fourier Transform Infrared-Ray (FTIR), X-ray Diffraction (XRD), Thermogravimetric (TGA), and Differential Scanning Calorimetry (DSC). From morphology study, the bundle fiber feature of CPBLH disintegrated into micro-size fibrils of CPMCC, showing the amorphous compounds were substantially removed through chemical depolymerization. Meanwhile, the elemental analysis also proved that the traces of impurities such as cations and anions were successfully eliminated from CPMCC. The CPMCC also gave a considerably high yield of 27%, which endowed it with great sustainability in acting as alternative biomass for MCC production. Physicochemical analysis revealed the existence of crystalline cellulose domain in CPMCC had contributed it 75.7% crystallinity. In thermal analysis, CPMCC had stable decomposition behavior comparing to CPBLH and CPAKL fibers. Therefore, Conocarpus fiber could be a promising candidate for extracting MCC with excellent properties in the future.

## 1. Introduction

Development of novel bio-based materials from waste biomass has become the focus of scientists and researchers to mitigate climate change. Agro-wastes are an abundantly available and inexpensive source of cellulose. The utilization of these biomasses is regarded as a dynamic approach that contributes to the establishment of a sustainable and cleaner environment [1,2]. *Conocarpus lancifolius* is a plant found natively in Somalia and Yemen. Today, it is widely cultivated across the Arabian Peninsula, North-East Africa, as well as some tropical coastal regions in North and South America [3,4]. It is commonly cultivated for landscaping and greening purposes in urban areas. Traditionally, Conocarpus had been used for medicinal and textile applications attributing to its anti-hypertension and anti-inflammatory properties. However, large agro-waste can be generated from Conocarpus plant due to improper management [5,6]. Conocarpus trunk fiber generally contains large amounts of lignocellulosic components, serving as the major cellulose source. This endows it with great potential for manufacturing cellulose derivative product such as microfibril cellulose and nanofibril cellulose.

Microcrystalline cellulose (MCC) is a depolymerized cellulose byproduct generated via the hydrolysis of inorganic acid. Generally, it possesses a length of more than 1 μm and diameter of 50–300 μm, as well as fascinating physical properties like stable thermal behavior, excellent reinforcing capability, and biocompatibility [7,8]. These have made it widely applicable in paper production as well as acting filler in composite materials. The thermal stability, particle size, and reinforcing capability of MCC depend significantly of the feedstock characteristics as well as the processing conditions such as acids concentration, acids type, hydrolysis conditions, and pre-treatment reactions [9]. MCC is employed as adsorbent, binder, and excipient in pharmaceutical and cosmetic industry. Moreover, in food and beverage industries, they act as additives, emulsifiers, stabilizers, thickeners, gelling agents, fat substitutes, suspending agents, and anti-caking agents. In addition, they are well known as reinforcing agents in composite production fields [10,11].

From literature studies, there is a lack of reports on the MCC isolation from Conocarpus fibers. Hence, this study aims to extract MCC from Conocarpus fiber through the chemical process of bleaching, alkali, and acid hydrolysis treatments. The obtained chemically treated fibers were examined for their morphology with microscopy and particle size analysis. Meanwhile, the functional chemistry, crystallinity, and thermal stability were also analyzed to comprehensively study the extracted fiber product.

## 2. Materials and Method 

### 2.1. Materials and Chemicals

Conocarpus fiber was collected from Riyadh, Saudi Arabia. The fiber was grinded into small pieces of about 0.5–1 cm and dried in oven for 24 hr. Chemical reagents of sodium hydroxide (NaOH), acetic acid (C_2_H_4_O_2_), sodium chlorite (NaClO_2_), and hydrochloric acid (HCl) were purchased from R & M Sdn. Bhd, Kajang, Malaysia. 

### 2.2. MCC Extraction

The grounded fiber was firstly treated with 500 mL of 2% NaClO_2_ (acidified with 5 mL C_2_H_4_O_2_) for 2 hrs at 80 °C with constant stirring, and this experimental setting was found optimal to produce clearly white color fiber from Conocarpus with the great removal lignin compound following trial and fail approach. Then, the filtrate containing lignin was removed, and the solid NaClO_2_-treated fiber (CPBLH) residue was collected by filtration with distilled water through nylon cloth. After that, the fiber was treated with 500 mL of 5% NaOH for 5 hr at 80 °C in der to swell up both cellulose and hemicellulose components which could aid in the following acid hydrolysis for depolymerization process. The collected NaOH-treated fiber (CPAKL) residue was neutralized with distilled water to pH 7 through nylon cloth until showing white color. In further acid hydrolysis treatment, the process was carried out at 80 °C for 30 min using 2.5 M of HCl solution with the aim to depolymerize the fiber into smaller particles. The resulting solution was then quenched for its acidic reaction with 10 times of cold distilled water and subsequently neutralized to around pH 3 through centrifugation. Finally, the acid-treated fiber (CPMCC) with soft pulpy structure was obtained after filtration and drying. 

### 2.3. Characterization 

#### 2.3.1. Functional Chemistry Analysis 

The functional chemistry of fiber samples was studied with Thermo Nicolet Nexus 670 FTIR analyzer (Thermo Scientific, Woodland, CA, USA) in the range of 400–4000 cm^−1^ wavenumbers, and resolution was maintained at 4 cm^−1^. The fibers were pelletized after grinding with potassium bromide (KBr) before analysis. 

#### 2.3.2. Crystallinity Analysis 

A PANalytical Empyrean X-ray diffractometer (XRD) (Malvern Panalytical B.V., Brighton, UK) was employed to examine the fibers with Cu Kα radiation. The X-ray generator was operating at 40 mA and 45 kV with slow scan rate of 2° per min, and the samples were placed on nickel coated steel holder. Crystallinity of fibers was calculated using the Segal method with Equation (1) as below:

(1) $$Crystalinity,\;CrI\;\left(\%\right)=\frac{\left(I_{mp}-I_{am}\right)}{I_{mp}}\times 100\%$$

where *I_mp_* refers to maximum point intensity at 22.7°, and *I_am_* refers to minimum point intensity at 18.50°, following reported work by French and Cintron [12]. 

#### 2.3.3. Morphology, Particle Size, Elements, and Yield Analyses 

Morphology was examined for the samples through JEOL, JSM-7610F Scanning Electron Microscopy (SEM) (JEOL Ltd., Tokyo, Japan) with 1–15 kV voltage ranges as the fibers were non-conductive. Before viewing, the fibers were coated with a metal palladium layer prior to loading onto the stub. Particle size analysis was performed for the fibers via ImageJ software. Moreover, elements were examined through Energy Dispersive X-ray (EDX) analysis under an acceleration voltage of 20 kV with working distance between 14.5–15.5 mm. Meanwhile, the yield (%) of fibers was determined with the given Equation (2) as below:

(2) $$Yield\;\left(\%\right)=\frac{\left(M_{2}-M_{3}\right)}{M_{1}}\times 100\%$$

where *M*_1_ is the mass of raw Conocarpus fibers; *M*_2_ is the total mass of treated-fiber in weighing container; *M*_3_ is the mass of weighing container. 

#### 2.3.4. Thermal Stability Analysis 

Thermal stability of fiber samples was assessed using a Thermogravimetric analyzer model TGA/SDTA 851e (Mettler-Toledo International Inc., Columbus, OH, USA). Thermal analysis was conducted in range of 30–900 ℃ with heating rate of 10 °C/min under nitrogen purge atmosphere, while Differential scanning calorimetry (DSC) was carried out at 30–600 ℃ at 10 ℃/min heating rate by using DSC 822 analyzer (Mettler-Toledo International Inc., USA).

## 3. Results and Discussion

### 3.1. Morphology and Elements Analysis

**Figure 1:**
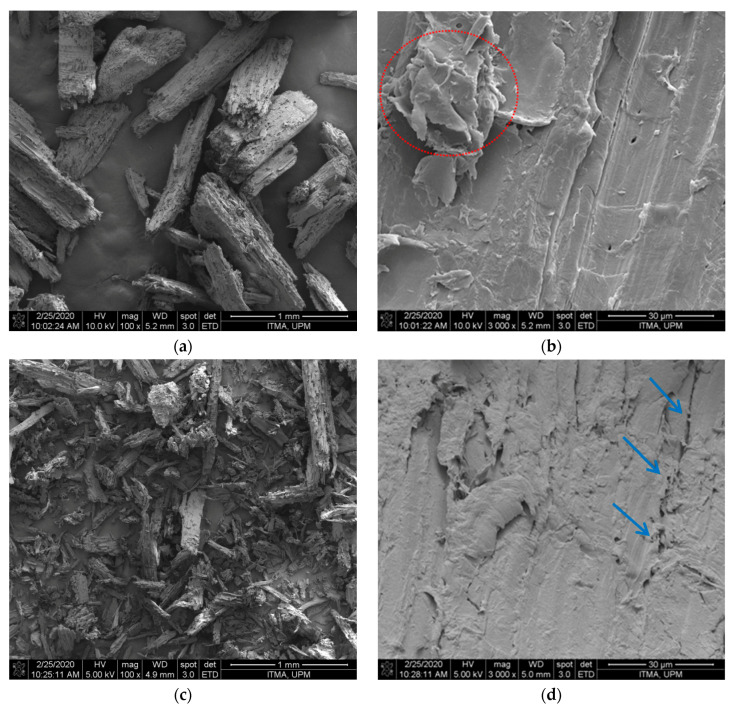

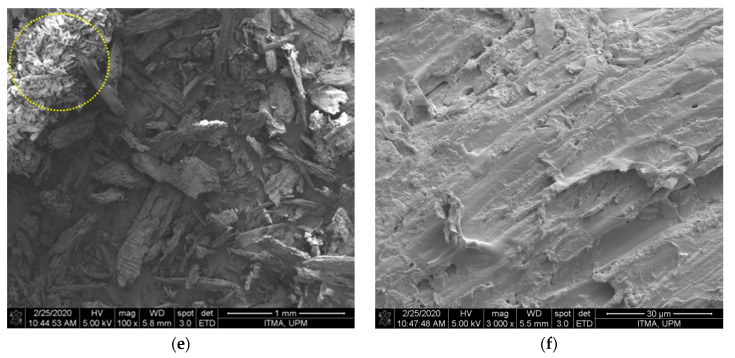
Micrographs of (**a**,**b**) bleached fibrefiber (CPBLH), (**c**,**d**) alkali-treated fibrefiber (CPAKL), and (**e**,**f**) microcrystalline cellulose (CPMCC) with magnifications of ×100 (**a**,**c**,**e**) and ×3000 (**b**,**d**,**f**) under SEM viewing.

Figure 1 presents the morphology of extracted fiber samples. CPBLH exhibited fairly smooth structure, indicating impurities of hemicellulose and lignin were substantially removed from the cellulose [13]. Meanwhile, the irregular shape of the bundle structure (red circle) was also observed for CPBLH fiber, further proving that the dissolution of fiber occurred in this bleaching treatment. For CPAKL, it showed a more ruptured and irrigated-like surface feature (blue arrow), evidencing the cellulose had undergone a swelling process during alkaline treatment [9,14]. Furthermore, CPMCC exhibited a smaller size structure compared to CPBLH and CPAKL. During acid hydrolysis treatment, hydronium ions penetrated the internal cellulose structure and broke down the weak amorphous part. Subsequently, depolymerization process separated the fiber into short fibril-like CPMCC product. However, CPMCC showed some aggregated particles (yellow circle), probably driven by the great surface area of MCC particles, which had higher tendency towards agglomeration [10,11]. In this work, the extracted CPMCC had a 60–110 μm diameter and 250–500 μm length. This could favor it with high surface reactivity for polymer fabrication process [1].

**Figure 2:**
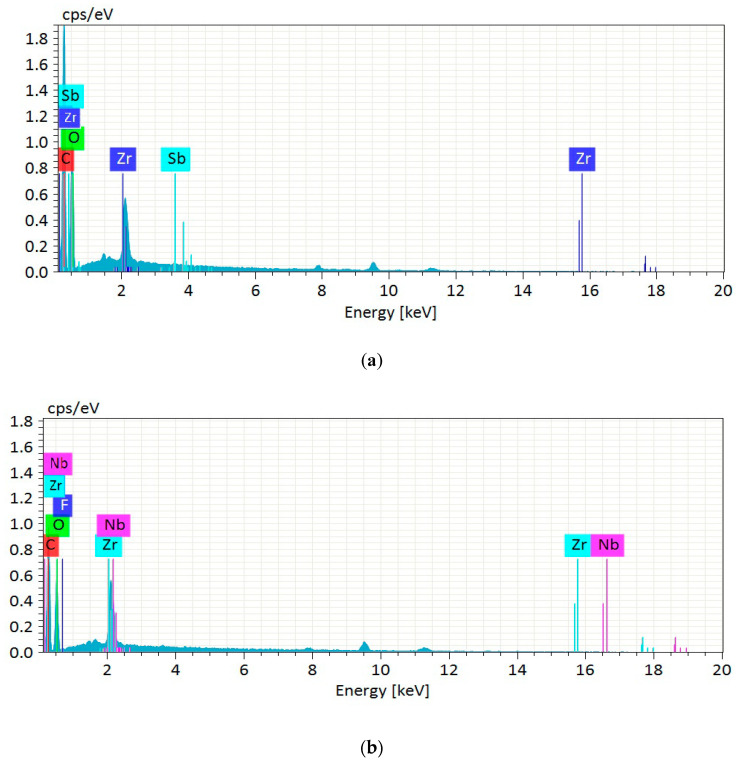

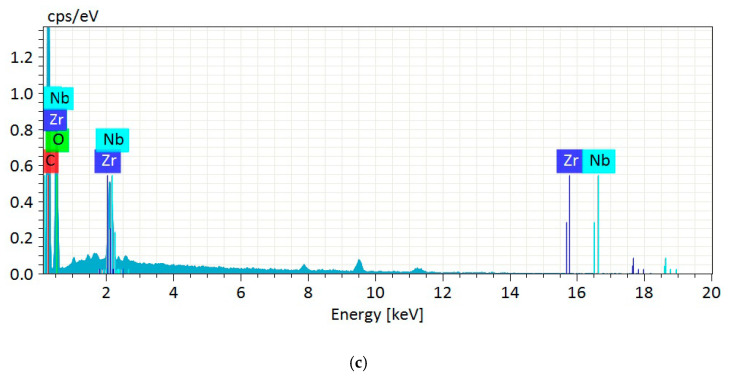
EDX spectra of (**a**) CPBLH, (**b**) CPAKL, and (**c**) CPMCC samples.

From EDX spectra (Figure 2), all samples revealed carbon and oxygen elements, which corresponded to the typical composition of cellulosic compounds. Meanwhile, there was unexpected presence of antimony and fluoride impurities for CPBLH and CPAKL, respectively. It was probably due to the unremoved residual compounds after alkaline and bleaching process that had accumulated the atomic ions through electrostatic attraction. Additionally, the detected zirconium and niobium in both CPAKL and CPMCC samples resulted from the metal coating effect during specimen preparation. As evaluated from Table 1, the built-up of antimony and fluoride impurities was extremely low for CPBLH and CPAKL after excluding zirconium and niobium elements. Moreover, the impurities were not found and could be negligible for CPMCC sample, implying the purity of cellulose content in CPMCC sample was high following acid hydrolysis treatment [2].

### 3.2. FTIR

**Figure 3:**
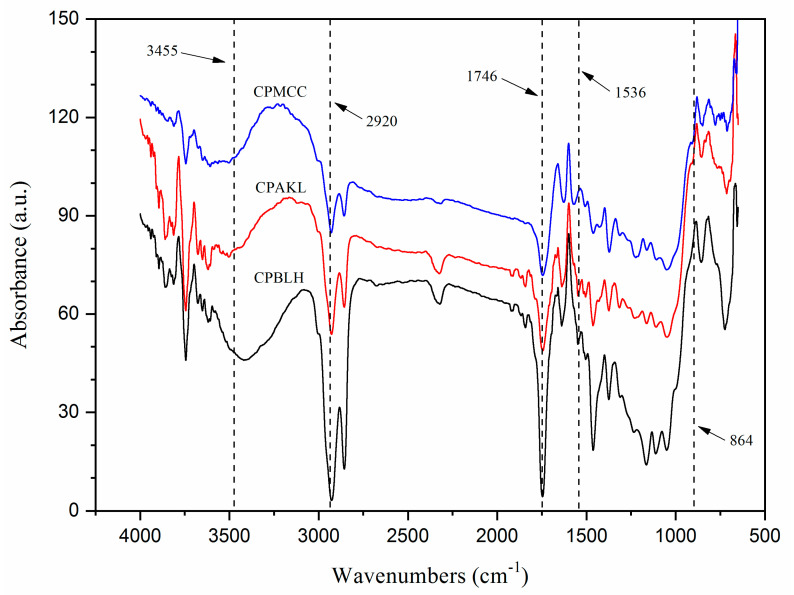
FTIR spectra of CPBLH, CPAKL, and CPMCC samples.

Figure 3 showed the FTIR spectrum of the samples. Each fiber revealed closely the same pattern of curves, indicating their functional groups were not significantly affected by the chemicals’ reaction [13]. The presence of absorption peaks at about 3400–3500, 2920, 1641, 1433, 1372, 1164, and 847 cm^−1^ were correlated to the typical functional groups of cellulosic samples [9,15]. Noticeably, the broad peak at 3408 cm^−1^ for CPBLH had shifted to 3469 cm^−1^ for CPAKL and, subsequently, to 3492 cm^−1^ for CPMCC, implying the increasingly exposed cellulose compartment throughout the treatments [11,14]. Moreover, another peak at 2920 cm^−1^ had reduced intensity for CPMCC. This was likely contributed by the presence of highly pure cellulose within the sample [16]. The peak intensities at 1746 cm^−1^ (C=O stretching of hemicellulose) and 1536 cm^−1^ (C=C vibration of lignin) were also noted decreasing for CPMCC, showcasing the process could remove substantial amorphous residual substances [1,17]. Meanwhile, the observed peaks at around 1641 and 1372 cm^−1^ were related to the cellulose–water interaction. The alteration of these peaks’ sharpness from CPBLH to CPMCC was probably influenced by the cellulose crystalline regions that had less affinity towards water absorption. This was supported by the remarkable change of peak intensities at about 1433 cm^−1^, which corresponded to the rearrangement of cellulose crystals [7]. In addition, the sharpness of peaks at 1164 cm^−1^ (C–O–C asymmetric vibration of ether groups) and 847 cm^−1^ (β-1,4-glycosidic linkages) were reduced possibly owing to the reorientation of cellulose order [2,18].

### 3.3. XRD

**Figure 4:**
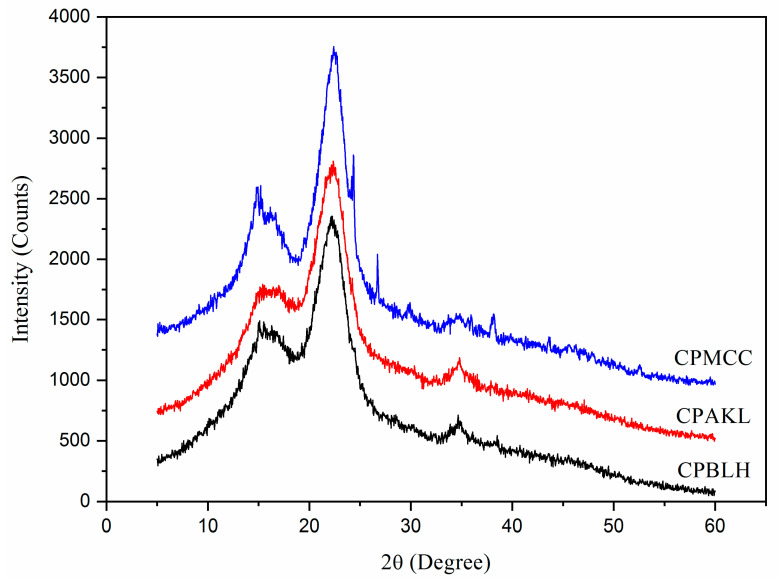
XRD curves of CPBLH, CPAKL, and CPMCC samples.

XRD patterns of fiber samples were shown in Figure 4. All fibers presented their main peaks at 2 theta of 15.4°, 16.9°, 22.7°, and 34.6°, which correlate to the (110), (200), and (004) crystal planes [12,19]. These peaks revealed that each fiber possessed Iβ type cellulose structure, evidencing the serial chemical processes did not change the fibers native structure [12,20]. The sharpest crystalline peak at about 22.7° was observed for CPMCC, implying it had the most stable cellulose structure among those samples [1,21]. From calculation, CPMCC possessed the highest crystallinity of 72.7%, followed by the CPBLH with 58.0%, and lastly by CPAKL with 54.9%. The lowest crystallinity for CPAKL was possibly associated to the alkaline cooking treatment that had softened the cellulose structure via swelling process. Besides this, the dramatically increased crystallinity for CPMCC could be explained by the gradually removal of amorphous components during acid hydrolysis, ultimately releasing highly rigid cellulose crystal structure [7,17]. Interestingly, another crystalline peak at 15.2° was noted prominently sharpening out for CPMCC fibers when compared to CPBLH and CPAKL. This might further prove that the acid hydrolysis played a role in eliminating weak residual substances such as surface binding components like lignin and hemicellulose to produce a greatly ordered crystalline cellulose structure [2,16]. Thus, the high crystallinity of CPMCC could endow it as potential load-bearing agent in composite reinforcement application.

### 3.4. Thermostability

**Figure 5:**
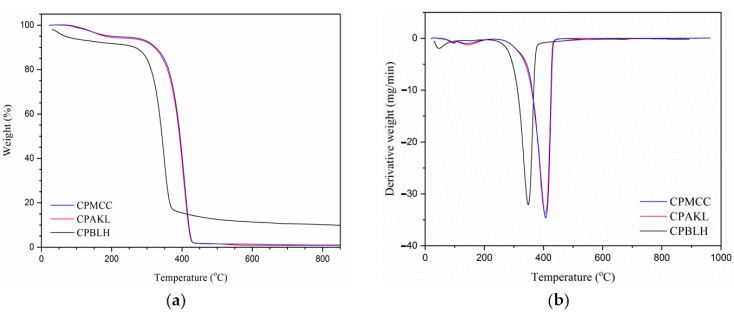
(**a**) TGA and (**b**) DTG curves of CPBLH, CPAKL, and CPMCC samples.

Figure 5 demonstrated the TGA curves of fiber samples. Initially, CPBLH showed a well reduced weight curve in the temperature range of 70–130 °C, which related to the evaporation of adsorbed water [22]. However, CPAKL and CPMCC fibers presented more likely a flattened curve, and the weight loss only became prominent in the 150–200 °C temperature range. This was probably due to the high cellulose contents in CPAKL and that CPMCC had absorbed a large amount of water. Consequently, it resulted in increased thermal capacitance degradation and delayed weight loss [15]. In the second degradation step, both CPAKL and CPMCC showed relatively higher decomposition temperature (TD) than that of CPBLH sample (Table 2). It was due to the presence of crystalline cellulose structure having greatly enhanced the thermal endurance towards high temperature [2]. Besides this, CPMCC only exhibited mildly improved decomposition temperature compared to CPAKL. It was possibly due to their same cellulose crystals order as aforementioned having contributed them similar thermal stability trend [16]. Additionally, in this work, the final char residue formation (WCR) was found remarkably low or both CPAKL and CPMCC with less than 2%, whereas the highest was found for CPBLH with more than 7%. This could evidence the exceptionally pure cellulose was obtained at the end of the chemical process [19]. Moreover, from DTG analysis, the maximum decomposition temperature (TMD) was the highest for CPMCC sample at 408.5 °C, suggesting its suitability to be applied in extreme temperature processing application.

**Figure 6:**
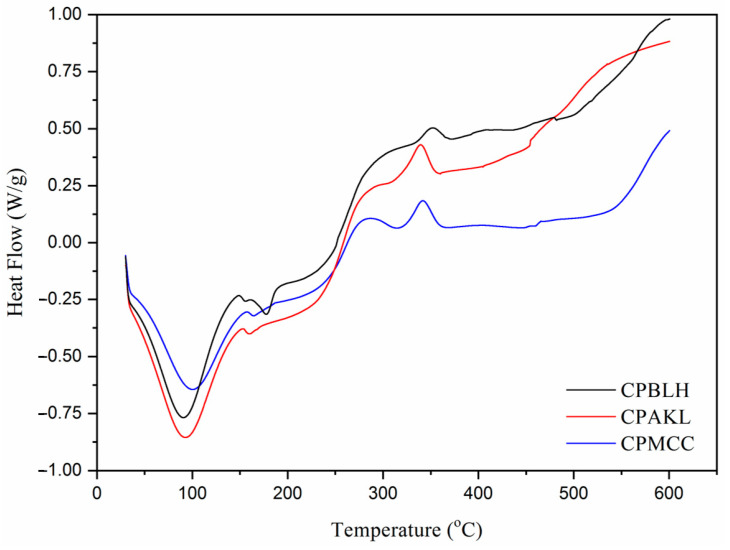
DSC curves of CPBLH, CPAKL, and CPMCC samples.

As for DSC analysis (Figure 6), an initial broad endothermic band from 60 °C to 140 °C was observed for each sample, where heat energy was absorbed to volatize the water molecules [12,20]. In the region of 150 °C to 200 °C, a small endothermic peak was noticed for CPAKL and CPMCC at 160.5 °C and 164.5 °C, respectively, whereas this peak was less prominent reflected by CPBLH at 154.9 °C and an additional larger peak was presented at 175.3 °C herein. This was probably owing to the containing of amorphous compounds which distorted the heat flow behavior in CPBLH [9]. Beyond 250 °C, CPMCC significantly revealed an exothermic peak near to 288.6 °C temperature. However, CPBLH (at 283.3 °C) and CPAKL (282.9 °C) are more likely to exhibit the exotherms as shoulder bands rather than as sharp peaks. This could indicate that the cellulose degradation process is more uniform in CPMCC as compared to CPBLH and CPAKL fibers [7,14]. Apart from that, another exothermic peak that occurred in the region of 340–360 °C was noted for all fibers, which related to the decarboxylation and depolymerization of cellulose [19]. 

## 4. Conclusions

The findings of the present study proved that the sequence of alkaline cooking, bleaching, and acid hydrolysis process is able to isolate Conocarpus fiber for MCC production with excellent properties. A morphology study revealed the MCC had a rough feature and a fibril shape feature which could provide it with high surface reactivity for composite fabrication. Meanwhile, the functional chemistry was not modified throughout the entire chemical treatment, while only lignin and hemicellulose substances were substantially removed. The crystallinity relating to the rigid structure of MCC was greatly improved, suggesting its suitability to be applied as load-bearing agent. Regarding thermal decomposition, the crystal domain of cellulose played a major role in withstanding the MCC towards high temperature. Hence, it was concluded that the MCC product isolated from Conocarpus trunk fiber is a sustainable way to develop a green composite in the future.

## Figures and Tables

**Table 1:** Elements composition and yield of fiber samples.

Samples	C (%) ^a^	O (%) ^b^	Sb (%) ^c^	F (%) ^d^	Zr (%) ^e^	Nb (%) ^f^	Yield (%)
CPBLH	64.71	34.51	0.02	-	0.75	-	52
CPAKL	64.76	32.48	-	0.91	1.08	0.77	41
CPMCC	66.62	32.37	-	-	0.62	0.40	27

^a^ Carbon; ^b^ Oxygen; ^c^ Antimony; ^d^ Fluoride; ^e^ Zirconium; ^f^ Niobium.

**Table 2:** Thermal analysis data of CPBLH, CPAKL, and CPMCC.

Samples	TD (°C) ^a^	TMD (°C) ^b^	WL (%) ^c^	WCR (%) ^d^
CPBLH	309.5	349.8	89.6	7.4
CPAKL	367.3	402.8	96.0	1.3
CPMCC	372.9	408.5	96.4	1.1

^a^ Decomposition temperature; ^b^ maximum decomposition temperature; ^c^ weight loss; ^d^ weight of char residue formation.
